# Magnetic domain interactions of Fe_3_O_4_ nanoparticles embedded in a SiO_2_ matrix

**DOI:** 10.1038/s41598-018-23460-w

**Published:** 2018-03-23

**Authors:** J. A. Fuentes-García, A. I. Diaz-Cano, A. Guillen-Cervantes, J. Santoyo-Salazar

**Affiliations:** 1 UPIITA-Instituto Politécnico Nacional, 07340 Ciudad de México, Mexico; 2 Departamento de Física, Centro de Investigación y de Estudios Avanzados del Instituto Politécnico Nacional, CINVESTAV-IPN, Av. IPN 2508, Zacatenco, 07360 Ciudad de México, Mexico

## Abstract

Currently, superparamagnetic functionalized systems of magnetite (Fe_3_O_4_) nanoparticles (NPs) are promising options for applications in hyperthermia therapy, drug delivery and diagnosis. Fe_3_O_4_ NPs below 20 nm have stable single domains (SSD), which can be oriented by magnetic field application. Dispersion of Fe_3_O_4_ NPs in silicon dioxide (SiO_2_) matrix allows local SSD response with uniaxial anisotropy and orientation to easy axis, 90° <001> or 180° <111>. A successful, easy methodology to produce Fe_3_O_4_ NPs (6–17 nm) has been used with the Stöber modification. NPs were embedded in amorphous and biocompatible SiO_2_ matrix by mechanical stirring in citrate and tetraethyl orthosilicate (TEOS). Fe_3_O_4_ NPs dispersion was sampled in the range of 2–12 h to observe the SiO_2_ matrix formation as time function. TEM characterization identified optimal conditions at 4 h stirring for separation of SSD Fe_3_O_4_ in SiO_2_ matrix. Low magnetization (M_s_) of 0.001 emu and a coercivity (H_c_) of 24.75 Oe indicate that the embedded SSD Fe_3_O_4_ in amorphous SiO_2_ reduces the M_s_ by a diamagnetic barrier. Magnetic force microscopy (MFM) showed SSD Fe_3_O_4_ of 1.2 nm on average embedded in SiO_2_ matrix with uniaxial anisotropy response according to Fe^3+^ and Fe^2+^ electron spin coupling and rotation by intrinsic Neél contribution.

## Introduction

The addressable Fe_3_O_4_ NPs in functionalized systems are of interest for the development of applications in magnetic hyperthermia, drug delivery and diagnosis agents and alternative cancer treatments due to their high biocompatibility^1–3^, bioactivity^4,5^, driving accumulation^6,7^ and magnetic excitation^8–10^ features. The systems based on superparamagnetic NPs behaviour have either biological or technological potential applications. Fe_3_O_4_ NPs have been extensively studied due to their interesting properties, which allow their evaluation and potential application as catalytic agents^11–13^, gas sensors^14^, water treatment agents^15^, environmental remediation agents^16–18^ magnetic resonance contrast agents^1,19^, ferrofluids^20^, data storage devices^21^, and electronic devices^22^. Superparamagnetic Fe_3_O_4_ NPs with SSD have crystal anisotropy in the order 1.1, which allows easy reorientation and energy exchange to generate magnetic hyperthermia in presence of a magnetic field AC^23^. However, avoiding Fe_3_O_4_ NPs agglomeration due to interactions between neighbours is a challenge for biological applications. One alternative is separating the Fe_3_O_4_ SSD considerable distances by embedding them in a negatively charged, amorphous SiO_2_ matrix^24^. This method allows a local response orientation of SSD to be obtained, according to Majetich S. A. *et al.;* if neighbouring superparamagnetic particles with a low anisotropy are coupled by exchange or dipolar interactions, their optimal separation for the maximum coercivity will allow the configuration of individually addressable SSD^25^. The local interactions will be the same, if the Fe_3_O_4_ particle size is similar. Then, SSD will have coherent rotation and contribute to the effective energy exchange by hyperthermia.

Current implementation of these systems depends on the superparamagnetic particle size (<20 nm) and SSD anisotropy, which directly influence the stability, dissipated energy and magnetic properties as function of time, such as the magnetic susceptibility and coercive field^26^. For this reason, different investigations have developed different methods to stabilize Fe_3_O_4_ NPs while preserving the main SSD properties^27–29^. V Reichel *et. al*. showed that the magnetic properties of magnetite are dependent on Néel relaxation. These depend on the particle size, morphology and interparticle interactions as well as the temperature and time-scale of the measurement in Fe_3_O_4_ NPs (9 ± 3 nm)^30^. The SiO_2_ media can be used to give Fe_3_O_4_ NPs specific accumulation in organs or tissues through bio-functionalization^31–35^ because it is an interaction-capable surface with ligands related to biological molecules.

Successful stabilization of Fe_3_O_4_ NPs with SiO_2_ can be achieved by using citrate as a dispersing agent. The polymerization time of SiO_2_ was considered in the range of 2–12 h. However, Fe_3_O_4_ functionalization and interaction media barriers with organic products can reduce M_s_. The magnetic response will depend on the volume, particle form and SSD population density in the system^36^. Rotation SSD Fe_3_O_4_ will depend on the intrinsic structure defined Néel interactions and magnetic moment distribution per volume unit by the easy axis crystal orientation, <100> or <111> and the coercivity terms, which are crucial for SSD uniaxial response^37^. The routes to control the dispersion Fe_3_O_4_ NPs embedded in SiO_2_ help to consolidate well defined the local SSD and their magnetic interactions. Superparamagnetic Fe_3_O_4_ NPs dispersions in an inorganic matrix allow the creation of a functional target or hyperthermia generators with potential applications in nanomedicine^38,39^.

## Results and Discussion

The key to obtaining Fe_3_O_4_ NPs is the ion relation ½ Fe^+2^: Fe^+3^ by co-precipitation and the additional dispersion reaction by Stöber route. These ions were dispersed with citrate and TEOS to produce Fe_3_O_4_ in an amorphous SiO_2_ matrix according to the methodology described by L. Yang *et al*.^40^ with some modifications.

**Figure 1:**
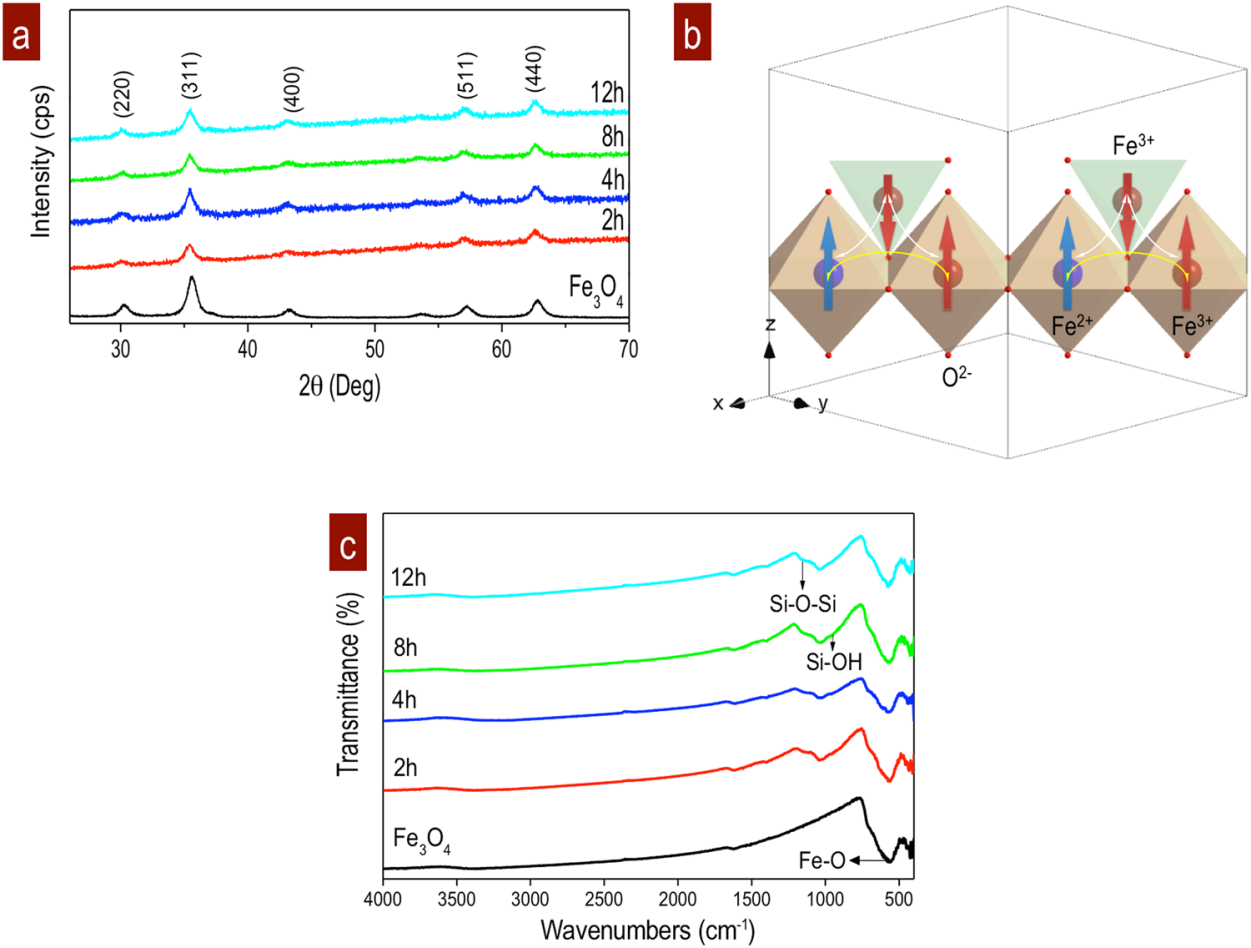
Structure and bonds of Fe_3_O_4_ NPs embedded in SiO_2_ matrix. (**a**) XRD shows the evolution with the stirring time; Fe_3_O_4_ NPs reference; Fe_3_O_4_ NPs+SiO_2_ matrix at 2, 4, 8, and 12 h. Background at these time is associated with the amorphous SiO_2_ matrix and small displacement of peaks due to Fe_3_O_4_ surface oxidation. (**b**) Ferromagnetic structure with octahedral sites of Fe^2+^ and Fe^3+^ contributes to the magnetic alignment of Fe_3_O_4_ NPs under magnetization. (**c**) FT-IR spectra of uncoated Fe_3_O_4_ NPs and those in the SiO_2_ matrix at 2, 4, 8 and 12 h of the coating reaction.

XRD results show the main crystalline reflections of Fe_3_O_4_ NPs with a cubic inverse spinel structure, forming a close packing *fcc* (*a* = 8.396 Å)^41^, Fig. 1a. Ferromagnetic spin interactions are defined by divalent Fe^2+^ and trivalent Fe^3+^ occupying octahedral sites and a double-exchange mechanism, while Fe^3+^ tetrahedral sites form an antiferromagnetic response, Fig. 1b. Because of superexchange, oxygen-mediated coupling, all the magnetic moments of the tetrahedral iron ions are aligned in a specific direction, while all the octahedral iron magnetic moments are aligned in the opposite direction^42^.

The spectra (Fig. 1a (2–12 h)) show the amorphous contribution of the SiO_2_ matrix and the Fe_3_O_4_ NPs, which do not lose their crystalline quality. Wide diffraction peaks from the Fe_3_O_4_ NPs are observed in an amorphous curve for the entire stirring range of 2–12 h. The fluorescence increased and the intensities decreased for each reflection in the patterns corresponding to the Fe_3_O_4_ NPs embedded in the SiO_2_ matrix. This is attributed to the confinement of the Fe_3_O_4_ NPs inside the amorphous SiO_2_ matrix.

Additionally, wide peaks are related to the nanometric particle size. Some changes in the NPs size were observed as a function of the reaction time, Fig. 1a. Supplementary information (s1) shows the FWHM values and sizes obtained through a modified Scherrer analysis by using a pseudo-Voigt function^43^. In some cases, the size of the particle increased due to agglomeration and the dynamic stirring energy. When the SiO_2_ matrix does not complete the polymerization, the uncoated Fe_3_O_4_ NPs form aggregates and reassemble^30^. Peaks of smaller particle sizes were defined after the stabilized reaction of the NPs in the SiO_2_ matrix with 4 h of processing. Average size in Fe_3_O_4_ NPs was 16.73 nm and *a* = 8.394 Å. Average sizes and lattice parameters in Fe_3_O_4_ NPs+ SiO_2_ matrix were (a) 16.49 nm and 8.360 Å at 2 h, (b) 15.37 nm and 8.3641 Å at 4 h, (c) 16.92 nm and 8,3619 Å at 8 h, and (d) 17.07 nm and 8.3614 Å. The lattice parameters showed small distotions as Fe_3_O_4_@*γ*-Fe_2_O_3_ by surface NPs oxidation.

The FT-IR spectra of different samples are presented in Fig. 1c. The interactions of the functional group Fe_3_O_4_ NPs in the SiO_2_ matrix can be observed. Characteristic iron-oxygen bond (Fe-O) signals at approximately 585 cm^−1^ are in the five spectra. Crystalline Fe_3_O_4_ is conserved after the embedding process. Also, it is possible to identify the SiO_2_ contributions in Fig. 1c; a broad band centred at 1048 cm^−1^ is observed. There are also two interactions of interest at 1113 cm^−1^, which is attributed to the Si-O-Si bonds, and another at 986 cm^−1^, which is due to the Si-OH groups from the matrix. These interactions slightly increased in intensity with the reaction time, which promoted the SiO_2_ layer thickness. No additional linkage interactions were observed for the functional groups characteristic of citrate and the stabilizing agents used during the experiment. These were completely removed during the particle washing step. This indicates that the reaction parameters, such as the concentration of the solutions and the reaction times, allow the dispersed particles to be obtained without adsorbed citrate molecules on the surface of the Fe_3_O_4_ NPs.

**Figure 2:**
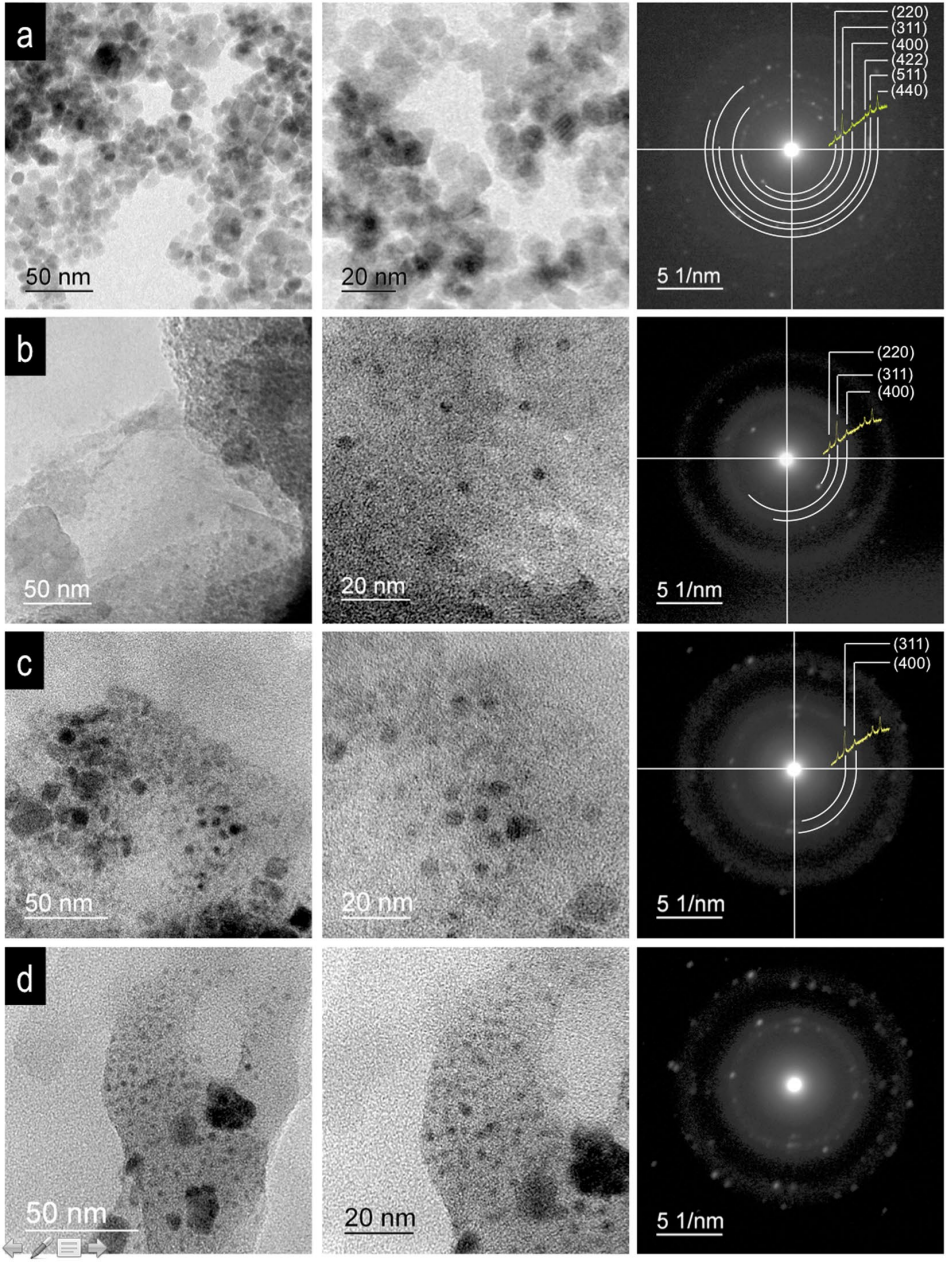
TEM images show the dispersion of Fe_3_O_4_ NPs at different reaction times in the SiO_2_ matrix: (**a**,**b**) Agglomeration of NPs at 2 h and (**c**) SAED with characteristic rings of the Fe_3_O_4_ NPs; the intensity depends on the population density of NPs; (**d**,**e**) individual SSD in the SiO_2_ matrix after 4 h and (**f**) SAED with dispersion in amorphous SiO_2_ plus Fe_3_O_4_ NPs; (**g**,**h**) SSD+agglomeration of NPs at 8 h, and (**i**) SAED with dispersion in amorphous SiO_2_; agglomeration increases the intensity of the electron diffraction; (**j**,**k**) SSD+agglomeration of NPs; (**l**) diffraction is increased by the groups of NPs agglomerates.

TEM results for Fe_3_O_4_ NPs embedded in SiO_2_ matrix as a function of time are shown in Fig. 2. The evolution of the processing was observed at 2, 4, 8 and 12 h of stirring: (1) Agglomeration of Fe_3_O_4_ NPs at 2 h, Fig. 2(a–c). Selected area of electron diffraction (SAED) results correspond to Fe_3_O_4_ structure with a low amorphous contribution from SiO_2_. (2) Good dispersion of individual Fe_3_O_4_ NPs at 4 h, Fig. 2(d–f). Under these conditions, the small, agglomerated nanoparticle entities are dispersed, and the TEOS polymerization is consolidated. SiO_2_ contributes a negative charge between the NPs to form individual SSD, Fig. 2f. SAED shows the contribution of Fe_3_O_4_ NPs and the amorphous matrix of SiO_2_. The crystalline Fe_3_O_4_ structure diffracted in the planes (220), (311) and (440). (3) The regions of agglomerated Fe_3_O_4_ NPs plus SSD dispersed for 8 h of stirring, Fig. 2(g–i). Under these conditions, there are NPs linked by electrostatic interactions and/or dipolar coupling. SAED shows the crystalline contribution of Fe_3_O_4_ NPs with diffraction planes in (311) and (440)^44^. The agglomerates increased in size. (4) Agglomerates plus isolated SSD were obtained in the SiO_2_ matrix after 12 h of stirring, Fig. 2(j–l). This phenomenon is associated with the strong interaction between neighbours. SAED shows the rings of the diffraction planes of Fe_3_O_4_, which are very similar in intensity to those in Fig. 2c. It could be said that the matrix of SiO_2_ formed at 4 h stirring is a negatively charged film that confines the SSD as individual entities. As the agitation time is increased to 8–12 h, the dynamic movement increases the coalitions between NPs and their interactions. As consequence, the attraction between NPs increases the agglomeration with a long agitation time.

**Figure 3:**
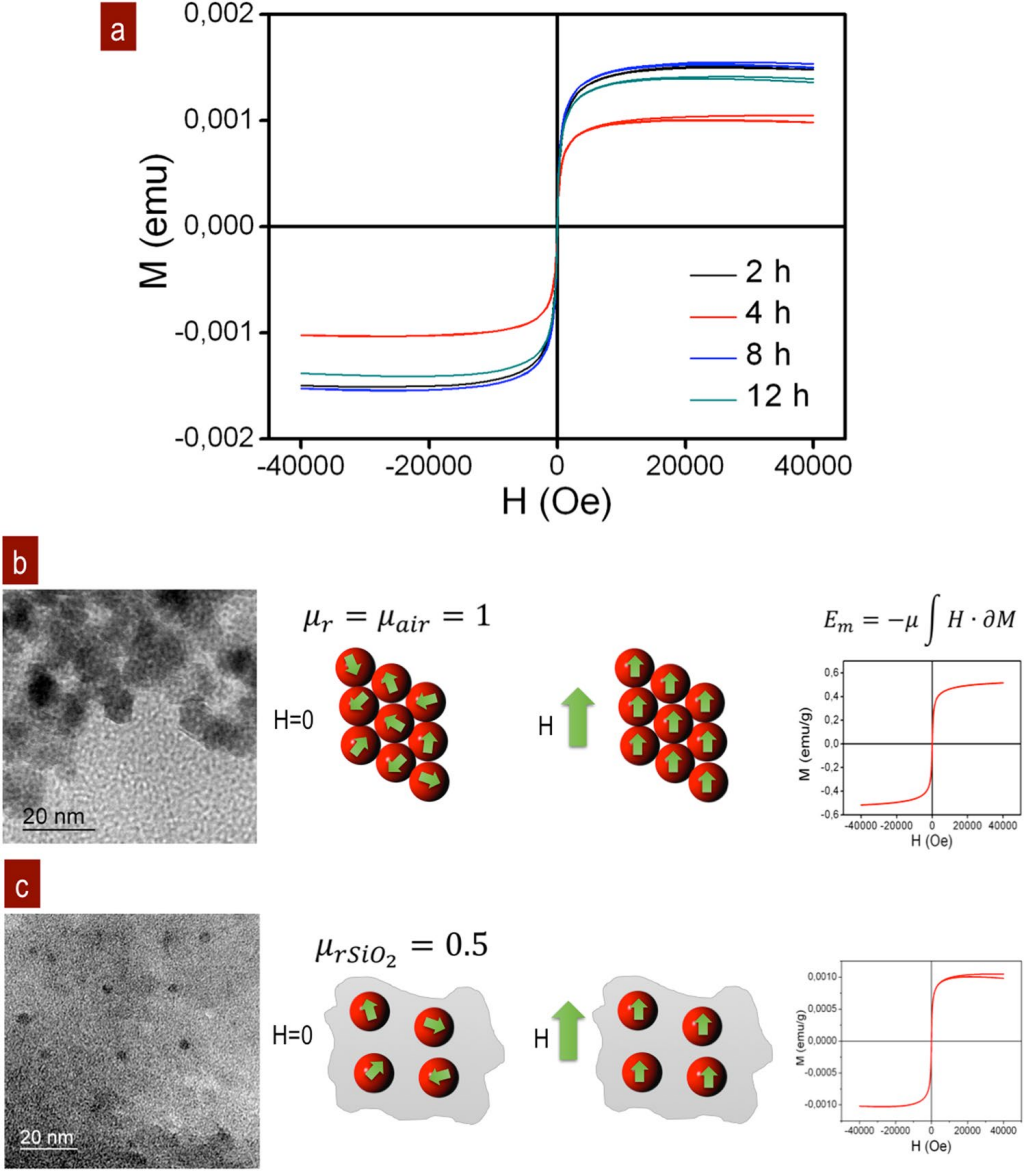
Magnetization responses from Fe_3_O_4_/SiO_2_: (**a**) H vs M as a function of the stirring time for well dispersed NPs as individual SSD at 4 h shows a lower magnetization. The amorphous SiO_2_ barrier between NPs reduces the magnification of 2, 4, 8, and 12 h of processing. Superparamagnetic signals as close loops were observed in all cases. (**b**,**c**) Differences in the magnetization due to the changes in the magnetic permeability. The magnetization saturation decreases with the *μ*_*r*_ of the SiO_2_ matrix media and the separation of NPs as SSD.

The main challenge in potential applications of Fe_3_O_4_ NPs lies in their superparamagnetic properties, uniaxial anisotropy, particle size and form. Typically, bulk Fe_3_O_4_ has a M_s_ of 91 emu/g, and its inner structure forms multidomains with magnetic anisotropic axial ratios larger than 5^45,46^. In superparamagnetic NPs, the magnetic anisotropic energy barrier is reduced and allows the rotation of SSD from a spin-up state to spin-down state to readily rotate the magnetic spin direction on the easy axis^47^. The magnetization from Fe_3_O_4_ NPs embedded in a SiO_2_ matrix shows superparamagnetic, closed hysteresis loops^48^. As the particle size of Fe_3_O_4_ decreased below the superparamagnetic diameter, M_s_ decreases as well, and the magnetic fluctuation leads to a small H_c_ near zero with a low remanence at 300 K^49^. Samples as a function of the stirring time were M_s_ = 0.0015 emu, H_c_ = 26.480 Oe at 2 h; M_s_ = 0.0010 emu, H_c_ = 24.000 Oe at 4 h; M_s_ = 0.0013 emu, H_c_ = 24.800 Oe at 8 h; and M_s_ = 0.0015 emu, H_c_ = 25.570 Oe at 12 h, Fig. 3a. Additionally, the TEM image showed the separation of the Fe_3_O_4_ NPs SSD embedded in the SiO_2_ matrix at 4 h of stirring. These parameters and the particle size are directly related to the descending H_c_ value as follows: (a) agglomerations of NPs 11.70 nm on average at 2 h; (b) fragmentation and dispersion of 5.64 nm NPs with distances from 5.54 to 29.21 nm as individual SSD in SiO_2_ at 4 h; (c) aggregates of 15.45 nm NPs on average and individual 4.47 nm NPs on average separated by 3.54 nm to 6.00 nm in SiO_2_ at 8 h; and (d) 13.7 nm average agglomerates and 6.02 nm NPs on average separated by distances 5.20 nm to 6.43 nm in SiO_2_ at 12 h. Coherent results were obtained by M_s_ and H_c_ with 4 h of stirring. This means that individual SSD have uniaxial anisotropy and are addressable by the separation between the NPs, Fig. 3c. However, in case of the polydispersion of NPs, M_s_ and H_c_ increase due to the major volume contribution from the neighbouring interactions and thermal fluctuations. The diamagnetic SiO_2_ barrier around the Fe_3_O_4_ NPs reduces M_s_. However, separation between the monodispersed NPs maintains the H_c_ as local SSD. These effects can also be observed in encapsulated Fe_3_O_4_ NPs with organic chains. Some organic coating media have important roles due their permeability, specificity and individual drive of the magnetic domains that can be accumulated in tumours^50^. The integration of SSD as functionalized systems at optimal distances and relatively high H_c_ values would increase the hyperthermia efficiency by magnetic field AC excitation in the frequency range 100 kHz–500 MHz^51^.

**Figure 4:**
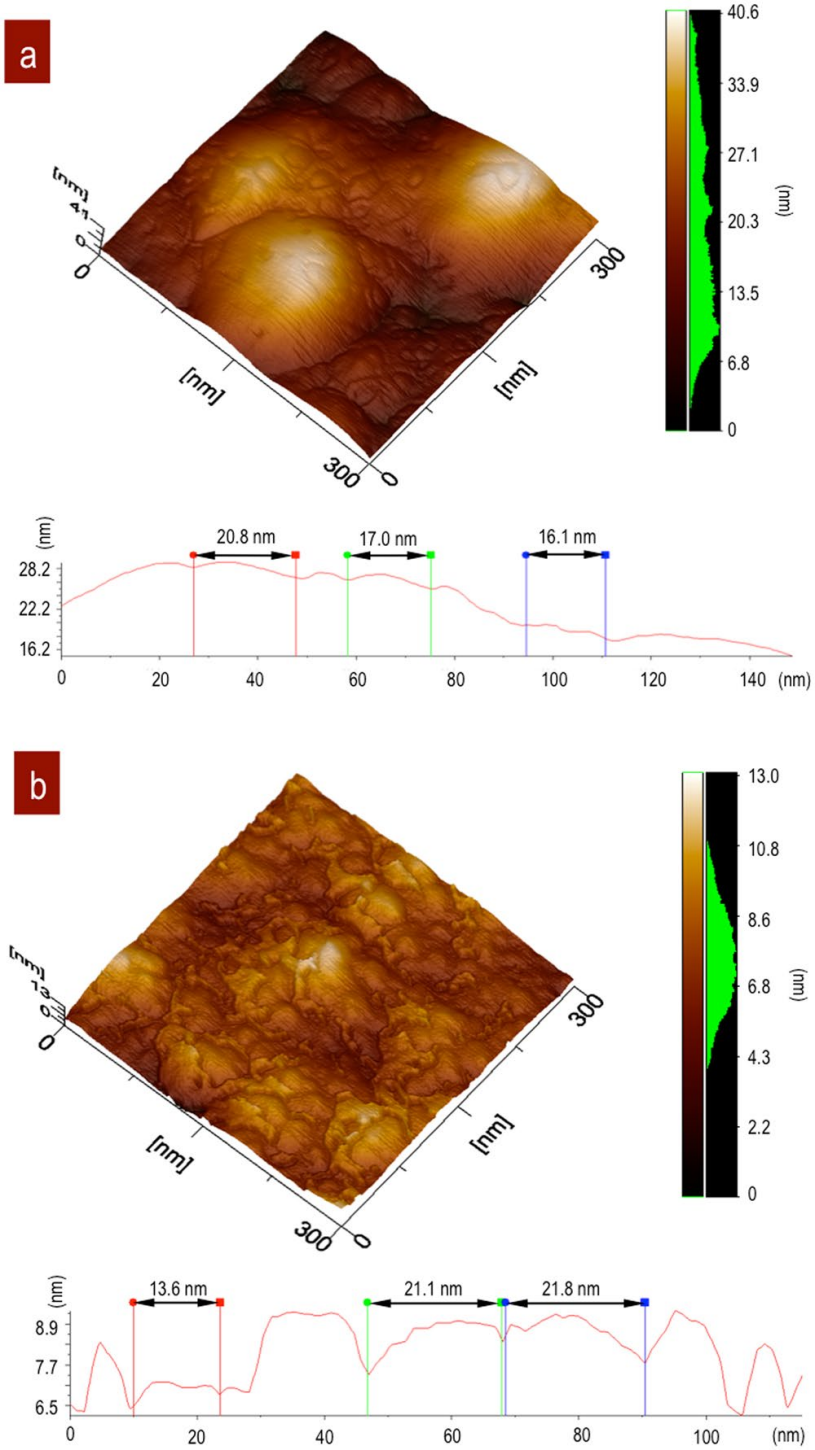
Topography by AFM. (**a**) Fe_3_O_4_ NPs immersed in a SiO_2_ matrix after 4 h of stirring. (**b**) 12 h of stirring with a high concentration of NPs. Profiles show isolated Fe_3_O_4_ NPs signals from (**a**) SSD at 4 h and agglomeration at 12 h (**b**).

Fe_3_O_4_ NPs distribution embedded in the SiO_2_ matrix were observed by atomic force microscopy. The 3D images show the differences in the dispersed nanoparticles according the stirring time for 4 and 12 h. Figure 4. The sample obtained with 4 h of stirring had local, isolated particles in the SiO_2_ matrix, Fig. 4a. The topography profiles show an estimated particle size of 6 nm and roughness of MRS = 4.53 nm. The images with 12 h of stirring showed 21.8 nm agglomerated particles and their neighbouring interactions with an MRS = 3.07 nm, Fig. 4b. The profiles below 3D images are associated to particle size and their agglomerations in both cases.

**Figure 5:**
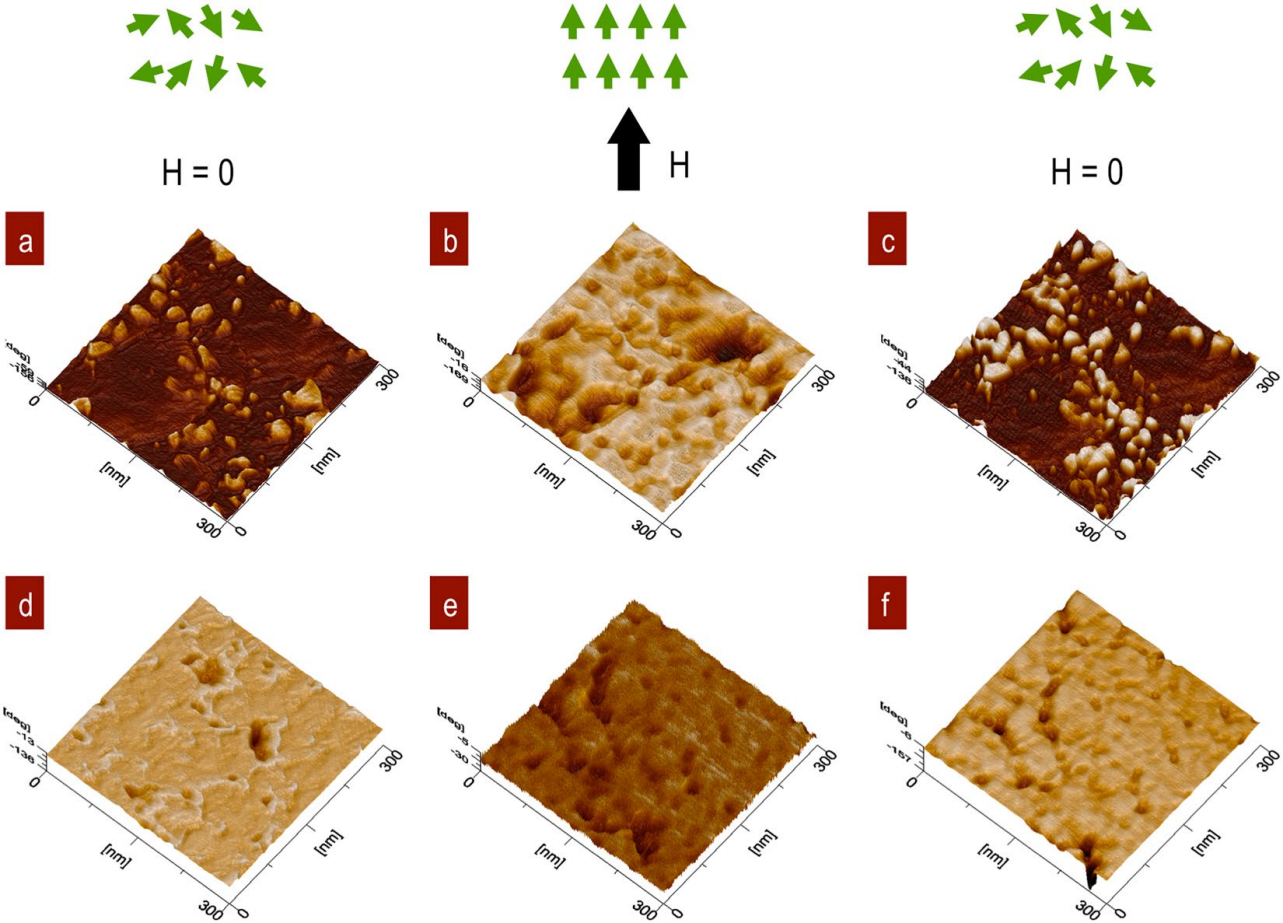
Magnetic domain interactions for the magnetization and demagnetization of (**a**) Fe_3_O_4_ NPs immersed in a SiO_2_ matrix after stirring for 4 h, H = 0; (**b**) orientation of the SSD at H = 12000 Oe; (**c**) at H = 0, the sample showed the local SSD orientation under the saturation conditions of the applied field; (**d**) agglomeration of NPs in a SiO_2_ matrix after 12 h at H = 0; (**e**) orientation of the NPs at H = 12000 Oe; and (**f**) disorder of the NPs at H = 0.

MFM shows the magnetic moment density per volume unit. The 3D images are generated by the interaction of magnetic tip polarization, which is normal with respect to the NPs surface, and sensing of the attractive and repulsive states^48^. SSD are NPs that can be aligned in parallel by exchanging forces of the electron spins from applied magnetic field. Inner magnetic structure Fe_3_O_4_ SSD with Fe^+3^ and Fe^+2^ electron spin coupling respond uniaxially because magneto-crystalline anisotropy forces tend to align in a preferred direction based on the easy axis. For superparamagnetic sizes, the relative magnitude of the boundary energy becomes larger than the magnetostatic energy. At a critical size, the spin direction can be oriented by exchange interactions in the SSD^37^. MFM 3D images showed surface of NPs by magnetization H↑ and demagnetization H = 0 effects from Fe_3_O_4_ SSD embedded in the SiO_2_ matrix. The magnetic interaction at the tip in the lift mode shows the disorder from the intrinsic structure at H = 0. Under the saturation measurement conditions H~1200  Oe for the Co-Ni tip, the SSD uniaxial orientation of the magnet is shown with the domains oriented at 90° for the majority *n*↑ and minority *n* ↓ states with H↑^48^. Finally, domain disorder is observed by demagnetization under the initial conditions H = 0. When the magnetic field AC is applied to these systems, the magnetic domains will fluctuate with local vibrations and will transform this energy into thermal energy. The orientation was observed in a non-continuous media with local SSD interactions at 4 h of stirring, Fig. 5(a–c). In the case of NPs agglomeration, the surface interactions showed a global response by addition of all magnetic domains in the 12 h stirring sample, Fig. 5(d–f).

**Figure 6:**
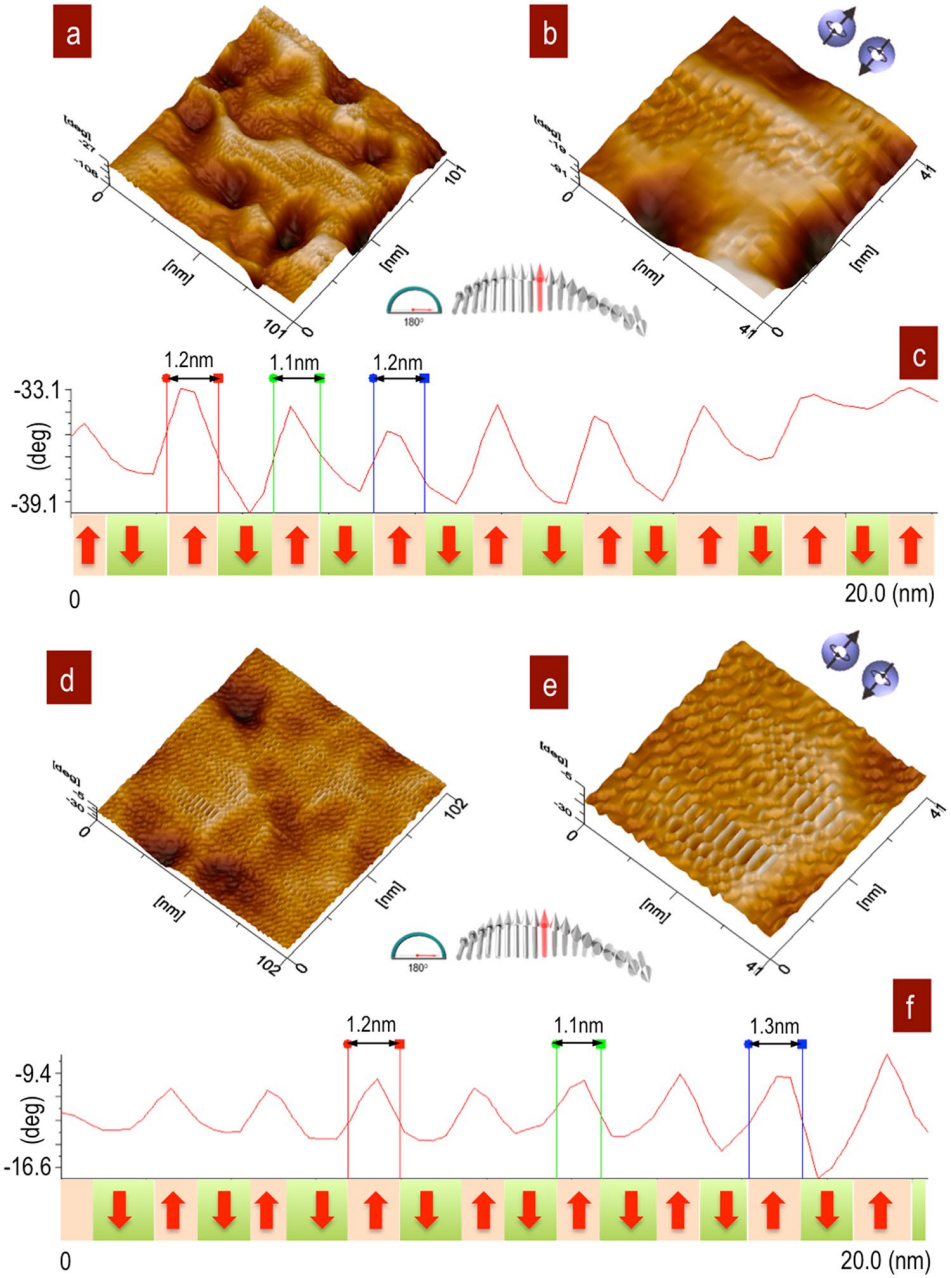
Parallel orientation of magnetic domains with uniaxial behaviour. (**a**) Fe_3_O_4_ SSD immersed in a SiO_2_ matrix after stirring for 4 h show desirable areas with stripes in the same direction, and the profile indicates the orientation of SSD, 90°; (**b**) after stirring for 12 h, the sample shows oriented magnetic domains and assembly by neighbouring interactions; profiles show the sum of all local SSD.

Zoom was performed under the saturation conditions H↑ to show the uniaxial behaviour. Parallel stripes from attractive and repulsive interactions define the magnetic domains. The exchange forces of the parallel alignment are result of the internal magnetic structure in Fe_3_O_4_ NPs, which are defined by Neél interactions, Fig. 6. The profiles show the *θ* (deg) and domains oriented in the normal direction, 90°, with average domain sizes of 1.2 nm and uniaxial anisotropy from main crystalline direction^37^. The anisotropy energy is given by *E*_*a*_ = KVsin^2^*θ*, where *K* is a typical constant of the material, *V* is the volume of the particle, *θ* is the angle between the magnetization and the easy axis. The local response of Fe_3_O_4_ NPs showed magnetic domains with an average size of 1.2 nm surrounded by the SiO_2_ matrix with stirring for 4 h. The profile defines the parallel interactions of the uniaxial response, Fig. 6a–c. However, the neighbouring interactions of Fe_3_O_4_ NPs in SiO_2_ matrix with 12 h of stirring show well-defined interactions in the same direction as the sum of local interactions with similar magnetic domains in the range of 1.2 nm, Fig. 6d,e. The Fe_3_O_4_ SSD in the SiO_2_ matrix show a local response of addressable superparamagnetic behaviour, which is mainly governed by particle size and shape of Fe_3_O_4_ cores in a monodispersion at magnetic field saturation. The degree axis in the profiles shows the deflection propensity in attraction and repulsion generated by H↑, and these values can change based on the roughness topography and H↑ intensity to form field lines between the tip and sample. The differences in the tip deflections from the SSD were −33.1°–(−39.1°) = 6.0° at 4 h of stirring, Figs. 6c, and −9.4°–(−16.6°) = 7.2° at 12 h of stirring, Fig. 6f, which are very similar. These results show the uniaxial responses of local SSD of individual NPs at 4 h and the sum of the SSD as a continuum in the NPs agglomeration at 12 h.

Tipically Hc decreases as a function of particle size in Fe_3_O_4_ NPs with close hysteresis loop. However, the superparamagnetic single-domains response of Fe_3_O_4_ NPs is maintained by local response SSD as agglomeration phenomena is reduced in the case of SiO_2_ matrix due to its permeability^47^. According to Dave S., R. Dave and G. Xiaohu, the formation of the domain walls is a process driven by the balance between the magnetostatic energy as a function of the particle size^52^.

## Conclusions

The synthesis strategy presented is a fast and effective option to avoid the agglomeration phenomenon of Fe_3_O_4_ NPs by stabilizing them in SiO_2_ as a function of the stirring time without altering their crystalline features.

The H_c_ is maintained in individual Fe_3_O_4_ SSD with uniaxial behaviour at 4 h of stirring. MFM showed SSD and that local NPs in the SiO_2_ matrix have similar responses as the NPs concentration increases. However, M_s_ is reduced by the distance between the NPs among the SiO_2_ matrix, and this depends on the permeability of the media. Neél interactions produce local fluctuations and energy transference from reorientable SSD by magnetic fields at high frequencies. The challenge is to control the Fe_3_O_4_ SSD as individuals in organic media to increase the response velocity and effective magnetic excitation to produce hyperthermia.

Fe_3_O_4_ NPs embedded in a SiO_2_ matrix are candidates as functional groups or specific organic linkers for cancer cell interactions and hyperthermia therapy. The development of dispersed SSD is mandatory to improve biological applications.

## Materials and Methods

For the synthesis, hydrochloric acid (HCl) (36.5–38.0%, BAKER ANALYZED ACS), deionized water (Millipore, 18.2 MΩ cm), iron chloride II (FeCl_2_, 98%), iron chloride III (97%), a tetramethyl ammonium solution (C_4_H_13_NO, 25 wt% in H_2_O), tetraethyl orthosilicate (TEOS, 98%), a 28% ammonium hydroxide solution (NH_4_OH) and absolute ethyl alcohol (C_2_H_5_OH) were all from Sigma-Aldrich and used as received without any purification.

### Synthesis of Fe_3_O_4_ NPs

The formation of nanostructured magnetite through a co-precipitation method^49^ was realized via the following: an aqueous solution of hydrochloric acid (HCl, 0.64 N) was prepared to dissolve the precursor salts of the Fe^+2^ (ferrous chloride, FeCl_2_) and Fe^+3^ ions (ferric chloride, FeCl_3_).

After the acidic solution was prepared, 6.25 mL was taken, and 2.52 g of FeCl_2_ was added. Another 25 mL of the solution was used to dissolve 6.99 g of FeCl_3_, and both solutions were kept under magnetic stirring for 1 h. Then, both solutions were mixed in a round-bottom, three-necked flask and bubbled with Ar for 5 min to remove any dissolved oxygen in the solution. The resultant solutions were stirred at 500 rpm and heated to 70 °C. Once the temperature stabilized, 21 mL of tetramethylammonium hydroxide was added dropwise, providing an alkaline environment for the formation of the magnetite. The solution turned black, which was an indication that iron oxide formed in the solution. After 40 min, the stirring and heating were stopped, and the mixture was allowed to cool to room temperature. The nanoparticles were washed with water and precipitated with the aid of a magnet until the pH of the dispersion was equal to 7. Once the nanoparticles were dispersed in water, they were freeze-dried for 12 h to obtain the magnetite powder.

### Synthesis of Fe_3_O_4_ in a SiO_2_ matrix

The Fe_3_O_4_ NPs powder (0.02 g) was taken and mixed with the same amount of citrate in an aqueous solution (250 mL) and mechanically stirred at 500 rpm for 1 h. Citrate was used as a dispersal agent to avoid agglomeration of the nanostructures prior to the formation of the SiO_2_ matrix.

Then, the SiO_2_ precursor solution was prepared as follows: 10 mL of water, 50 mL of ethanol, 5 mL of ammonium hydroxide and 0.2 mL of TEOS were mixed by magnetic stirring for 10 min. After that, the solution of magnetite and citrate was mixed with the SiO_2_ precursor solution. To modify the thickness of the SiO_2_ coating, the mixture reacted for 2, 4, 8 and 12 h under mechanical stirring at 500 rpm. Once the different times passed, the particles were washed with ethanol and precipitated with the help of a magnet. They were dried at room temperature, and the powder was characterized.

The analysis of the material allowed determination of the optimal reaction time conditions to reduce the agglomeration in the citrate suspension. Additionally, these samples were processed by the Stöber method to embed the magnetite NPs in the matrix of silicon dioxide. This is an easy methodology to reduce agglomeration and to improve the Fe_3_O_4_ NPs dispersal in the SiO_2_ matrix and the magnetic properties of the magnetite under different stirring conditions.

To determine the properties of the nanostructures, different characterization techniques were used: RIGAKU Smart Lab X-ray diffractometer, XRD, was used in the Bragg-Brentano configuration with 0.02 steps in the range from 25 to 70°, 2 *θ* with a copper source of 1.5424 Å and wavelength to define Fe_3_O_4_/SiO_2_ structures. PowderCell Software was used to analyse experimental diffractograms from XRD^53,54^. Their dispersion and particle sizes were observed by JEOL-JEM 2010 transmission electron microscope, TEM, with a LaB_6_ filament at an acceleration voltage of 200 kV. The functional groups of silicon and magnetite were examined with an FT-IR spectrophotometer, NICOLET 6700. The magnetic response was determined using a MPMS3 Magnetometer SQUID of Quantum Design with a sensitivity of 5 × 10^−8^ emu.

Magnetic force microscopy (MFM) was performed with a scanning probe microscope (SPM) JEOL JSPM 5200 multimodes. The samples were dispersed in carbon adhesive tape over the holder. An ultra-sharp silicon cantilever NSC14/Co-Cr/15 micro-mesh was exposed to a strong neodymium magnet. The voltage and lift conditions were defined according to the magnetic surface interactions of the sample with the tip lift output [0.030–0.1 V].

## Electronic supplementary material

Supplementary Information
