# Supplementary material for: Magnetic domain interactions of Fe3O4 nanoparticles embedded in a SiO2 matrix
Source: Sci Rep. 2018 Mar 23;8:5096. doi: 10.1038/s41598-018-23460-w (PMC5865143; doi:10.1038/s41598-018-23460-w)
Supplement: Supplementary file 1 — Supplementary Information [file 41598_2018_23460_MOESM1_ESM.docx]

| **FWHM** | **30^o^** | **35.53 ^o^** | **43 ^o^** | **53.57 ^o^** | **56.87 ^o^** | **62.67 ^o^** |
| --- | --- | --- | --- | --- | --- | --- |
| **Fe_3_O_4_** | 0.52 | 0.48 | 0.56 | 0.7 | 0.58 | 0.48 |
| **2h** | 0.59 | 0.4 | 0.6 | 0.66 | 0.58 | 0.56 |
| **4h** | 0.47 | 0.5 | 0.62 | 0.58 | 0.7 | 0.77 |
| **8h** | 0.5 | 0.49 | 0.58 | 0.65 | 0.6 | 0.46 |
| **12h** | 0.58 | 0.46 | 0.57 | 0.53 | 0.65 | 0.49 |

**Supplementary information (S1)**

**Magnetic domain interactions of Fe_3_O_4_ nanoparticles embedded in a SiO_2_ matrix**

J.A. Fuentes-García^1^, A.I. Diaz-Cano^1^, A. Guillen-Cervantes^2^, J. Santoyo-Salazar^2^^[[1]](#footnote-1)^*

^1^ UPIITA-Instituto Politécnico Nacional, 07340, Ciudad de México, México

^2^ Departamento de Física, Centro de Investigación y de Estudios Avanzados del Instituto Politécnico Nacional, CINVESTAV-IPN, Av. IPN 2508, Zacatenco, 07360, Ciudad de México, México

**Table 1.** FWHM of Fe_3_O_4_ nanoparticles embedded in SiO_2_ matrix from XRD at different time of stirring.

**Table 2.** Particle sizes from FWHM Fe_3_O_4_ nanoparticles embedded in SiO_2_ matrix from XRD in all diffracted peaks from 25-70 **^o^** 2(theta) at different time of stirring.

| **Sample** | **30^o^** | **35.53 ^o^** | **43 ^o^** | **53.57 ^o^** | **56.87 ^o^** | **62.67 ^o^** | **Average size (nm)** |
| --- | --- | --- | --- | --- | --- | --- | --- |
| **Fe_3_O_4_** | 16.53 | 18.16 | 15.93 | 13.28 | 16.28 | 20.25 | 16.73 |
| **2h** | 14.57 | 21.79 | 14.87 | 14.09 | 16.28 | 17.36 | 16.49 |
| **4h** | 18.28 | 17.43 | 14.39 | 16.03 | 13.49 | 12.62 | 15.37 |
| **8h** | 17.19 | 17.79 | 15.38 | 14.31 | 15.73 | 21.13 | 16.92 |
| **12h** | 15.91 | 18.95 | 15.65 | 17.55 | 14.52 | 19.84 | 17.07 |

1. * Corresponding author: jsantoyo@fis.cinvestav.mx, Tel: +(52) 555 747 38 00 x6756 [↑](#footnote-ref-1)
